# Prevalence and Associated Factors of Glaucoma in Rural Central India. The Central India Eye and Medical Study

**DOI:** 10.1371/journal.pone.0076434

**Published:** 2013-09-30

**Authors:** Vinay Nangia, Jost B. Jonas, Arshia Matin, Krishna Bhojwani, Ajit Sinha, Maithili Kulkarni, Rajesh Gupta, Anshu Khare, Shubhra Agarwal, Karishma Bhate, Prabhat Nangia, Purna Nangia, Songhomitra Panda-Jonas

**Affiliations:** 1 Suraj Eye Institute, Nagpur, India; 2 Department of Ophthalmology, Medical Faculty Mannheim of the Ruprecht-Karls-University Heidelberg, Mannheim, Germany; Wadsworth Center, United States of America

## Abstract

**Purpose:**

To assess the prevalence of glaucoma in rural Central India.

**Methods:**

The population-based Central India Eye and Medical Study is a population-based study performed in a rural region of Central India. The study included 4711 subjects (aged 30+ years). A detailed ophthalmic and medical examination was performed. Glaucoma was defined by glaucomatous optic disc morphology, and in a second step, by the criteria of the International Society of Geographical and Epidemiological Ophthalmology (ISGEO).

**Results:**

Optic disc photographs were available for 4570 (97.0%) subjects. Glaucoma was detected in 122 subjects (51 unilateral) (2.67% (95%CI: 2.20, 3.14). Glaucoma prevalence for the age groups of 30–39yrs, 40–49yrs, 50–59yrs, 60–69yrs, 70–79yrs, and 80+ years was 0.54% (95%CI: 0.11, 0.98), 1.03% (95%CI: 0.49, 1.57), 1.40% (95%CI: 0.58, 2.23), 6.62% (95%CI: 4.92, 8.31), 8.71% (95%CI: 5.55, 11.75), and 14.3% (95%CI: 4.13, 24.4), respectively. In multivariable analysis, glaucoma was associated with higher age (*P*<0.001), lower body mass index (*P* = 0.025), lower blood hemoglobin concentration (*P* = 0.03), higher intraocular pressure (*P*<0.001), disc hemorrhages (*P*<0.001), higher prevalence of myopic retinopathy (*P*<0.001), lower level of education (*P* = 0.03), longer axial length (*P*<0.001), thinner retinal nerve fiber layer (*P*<0.001), higher vertical cup/disc diameter ratio (*P*<0.001), and narrow anterior chamber angle (*P* = 0.02). Ratio of open-angle glaucoma to angle-closure glaucoma was 7.7:1 (1.93% (95%CI: 1.64, 2.22) to 0.24% (95%CI: 0.14, 0.34)). Using the ISGEO criteria, glaucoma prevalence was 2.8% (95%CI: 2.3, 3.3) with a less clear association with older age.

**Conclusions:**

Glaucoma prevalence in remote rural Central India is comparable to other regions. Associated factors were older age, lower body mass index, lower blood concentration of hemoglobin, lower level of education, higher intraocular pressure, disc hemorrhage, myopic retinopathy, and longer axial length. The ratio of open-angle glaucoma to angle-closure glaucoma was about 8:1.

## Introduction

Knowledge about the prevalence of a disease and the associated factors is instrumental for an improved detection of the disorder and for a better understanding of its pathogenesis. Factors associated with the development of glaucoma or with its further progression have been examined in several population-based studies and in numerous large hospital-based investigations [1]–[6], such as Ocular Hypertension Treatment Study [3], the Advanced Glaucoma Intervention Study [4], the Collaborative Initial Glaucoma Treatment Study [5], the Early Manifest Glaucoma Trial [6], and others. These studies suggested that high intraocular pressure, low blood pressure, low ocular perfusion pressure, narrow anterior chamber angles, thin corneas, pseudoexfoliation, a low body mass index, and myopia were some of the factors associated with glaucoma. These factors were examined in separate investigations so that inter-dependencies between some of the parameters could not be addressed. Also, most of the studies were performed in Western countries or in urbanized regions. In our study, we tried to include all of the aforementioned parameters to avoid confounding effects by neglected inter-relationships between the factors. We included patients with both, open-angle glaucoma and angle-closure glaucoma, and we performed our study in a population-based study design to avoid a potential referral bias inherent to any hospital-based study. Finally, we carried out our study in a remote rural region with a relatively low degree of technical development to get information on the prevalence of glaucoma and its associate factors at a stage before industrialization started.

## Methods

### Ethics statement

The Medical Ethics Committee of the Medical Faculty Mannheim of the Ruprecht-Karls-University Heidelberg and the Ethics Committee of the Suraj Eye Institute/Nagpur approved the study; all participants gave informed written consent, according to the Declaration of Helsinki.

The Central India Eye and Medical Study (CIEMS) is a population-based cross-sectional study performed in Central India between 2006 and 2008. As described in detail previously, the study was performed in 8 remote villages in Kalmeshwar Tehsil, a rural region of Eastern Maharashtra at a distance of about 40 km from Nagpur in the geographical center of India [7], [8]. The villages were chosen as locations for the study because they were located in a typical rural region of Central India, and were a relatively long distance from the nearest city (Nagpur). Of a total population of 13,606 villagers, 5885 subjects met the inclusion criterion of an age of 30+ years. There was no exclusion criterion. Of the 5885 eligible subjects, 4711 subjects (2191 men (46.5%)) participated, resulting in a response rate of 80.1%. The mean age was 49.5±13.4 years (median: 47 years; range: 30–100 years), and the mean reported monthly income was 1584±1233 rupees (1 US dollar equals roughly 50 rupees); the rate of illiteracy was 35%. Among the 1174 non-participants were 685 (58.3%) men; the mean age was 48.6±14.1 years (median: 45 years; range: 30–95 years). The group of study participants and the group of non-participants did not differ significantly in age (P = 0.06), while the proportion of men was significantly (P<0.001) higher in the group of non-participants.

All examinations were carried out at the hospital. Trained social workers filled out a questionnaire for the participants; this questionnaire included questions regarding socioeconomic background and living conditions, tobacco use and alcohol consumption, and any known diagnosis of major systemic diseases. In all subjects, the pulse, arterial blood pressure, and body height and weight were recorded. One-and-a-half hours after a standardized lunch, blood and urine samples were obtained and biochemically analyzed. A detailed ophthalmologic examination included testing of visual acuity by ophthalmologists or optometrists. Uncorrected visual acuity and visual acuity with the subjectś glasses and after refractive correction were measured using modified Early Treatment of Diabetic Retinopathy Study (ETDRS) charts (Light House Low Vision Products, New York, NY) at a distance of 4 meters. Automated refractometry and subjective refraction were performed on all subjects independent of visual acuity. Perimetric examinations were performed with frequency-doubling perimetry using the screening program C-20-1 (Zeiss-Humphrey, Dublin, CA). Intraocular pressure was measured by a slit lamp mounted Goldmann applanation tonometer; if the measurements were higher than 21 mmHg, tonometry was repeated. Slit lamp biomicroscopy was carried out by a fellowship-trained ophthalmologist, and any abnormality of the anterior segment was noted. With the subject in the supine position, corneal pachymetry and biometry were carried out via sonography using the Pacscan (Sonomed, Lake Success, NY). Central corneal thickness, anterior chamber depth, lens thickness and axial length were measured on each eye of all subjects. The examinations were performed up to five times each, and the mean values were taken. In subjects with poor visual acuity and poor fixation or in highly myopic subjects with posterior staphyloma, if the measurements varied, they were repeated until reproducible measurements were obtained. Using the slit lamp, photographs of the limbal region were taken to assess the limbal anterior chamber depth at the most peripheral part of the cornea, as described by van Herick [9]. With the slit lamp beam set at an angle of 60° to the sagittal axis, the chamber depth was expressed as a percentage of the corneal thickness at that location [10]. Gonioscopy was performed for all study participants in dim illumination using the magnaview single mirror gonio lens (Ocular Instruments, Bellevue, WA. USA). The slit beam was brought to its narrowest, and least height on a Haag Streit type slit lamp, to reduce the effect of light on the anatomy of the anterior chamber angle. The chamber angle was estimated as open, if in primary position the posterior pigmented part of the trabecular meshwork was visible without indentation [11], [12]. There was appositional closure of the angle if, independently of the direction of gaze, the posterior trabecular meshwork could be seen only upon indentation. Synechial closure of the anterior chamber angle was determined by indentation gonioscopy. In subjects with any extent of occludable angles, indentation gonioscopy was performed with the Sussman 4 mirror goniolens (Ocular Instruments, Bellevue, WA. USA). The pupil was dilated using tropicamide 0.8% and phenylephrine 5% three times at 15 minute intervals so that all subjects attained maximal pupillary dilatation. A second slit lamp examination was performed to assess the presence of pseudoexfoliation of the lens [13].

Digital photographs of the lens were taken and nuclear sclerosis was graded according to the Age Related Eye Disease Study [14]. Retro-illuminated photographs of the lens for assessment of cortical opacities were obtained using the Zeiss FF450 fundus camera (Zeiss Meditec Co., Oberkochen, Germany).

Digital photographs of the optic disc (20 degrees) and photographs centered on the macula and the optic disc (50 degrees) using the Zeiss FF450 fundus camera were taken. Magnification by optic media was corrected for by a built-in algorithm. Using the planimetric analysis system of the fundus camera, we outlined the margins of the optic disc and optic up on the digitized photographs and measured the area and horizontal diameter and vertical diameter of the optic disc and cup. Glaucoma was defined by a glaucomatous appearance of the optic disc. The optic nerve head was glaucomatous (1) if the inferior-superior-nasal-temporal (ISNT)-rule of the neuroretinal rim shape was not fulfilled in early glaucoma and in eyes with a normally shaped optic disc (i.e., the rim width at the inferior disc pole or at the superior disc pole was equal to or smaller than the rim width temporal; it included a notch in the neuroretinal rim in the temporal inferior region and/or the temporal superior region); or (2) if an abnormally large cup was present in a small optic disc which normally would not show cupping. In all eyes with glaucoma, the visibility of the retinal nerve fiber layer was locally and/or segmentally reduced. The assessment of the optic disc photographs was carried in a masked manner without knowledge of intraocular pressure or the perimetric results. Each photograph of a glaucomatous optic disc was independently adjudicated by two senior graders (VN and JBJ). In addition to the optic disc photographs, confocal laser scanning tomograms (HRT, Heidelberg Engineering, Heidelberg, Germany) of the optic disc were taken.

The whole glaucoma group was differentiated into subjects with open-angle glaucoma and with primary angle closure glaucoma. Open-angle glaucoma was characterized by an open anterior chamber angle, in addition to a normal depth of the anterior chamber as assessed by slit lamp biomicroscopy. In angle-closure glaucoma, the anterior chamber angle was occluded or occludable. The anterior chamber angle was defined as occludable, if ≥270° of the posterior trabecular meshwork could not be seen upon gonioscopy [11], [12], [15], [16]. In addition, other features for angle-closure glaucoma were iris whirling and glaukomflecken in the anterior subcapsular lens region, in combination with a narrow anterior chamber angle.

Fundus photographs were graded for signs of myopic retinopathy which was defined as the presence of at least 1 of the following features: staphyloma, lacquer cracks, Fuchs’ spot, or chorioretinal atrophy at the posterior pole [17]. Staphyloma was defined by localized ectasia of the sclera according to the classification used by Curtin [18]. Kinking of retinal vessels at the border of the ectatic region, complete loss of retinal pigment epithelium, and a loss of the choriocapillaris were the main morphologic features used to characterize a staphyloma. Myopic chorioretinal atrophy was defined as described by Steidl and Pruett [19].

Only those subjects with assessable optic disc photographs were included into the study. The status of the lens was no inclusion or exclusion criterion. Statistical analysis was performed using a commercially available statistical software package (SPSS for Windows, version 20.0, SPSS, Chicago, IL). Continuous data were presented as mean ± standard deviation. Chi-square tests were used to compare proportions. Logistic or linear regression models were used to investigate the associations of the presence of glaucoma with the continuous (e.g., intraocular pressure) or categorical outcomes (e.g., diabetic retinopathy). All *P*-values were 2-sided and were considered statistically significant when the values were less than 0.05. In the analysis, we first calculated the mean values in the study population. In a second step, we examined the association between the prevalence of glaucoma and other factors in univariate analysis. In a third step, we performed a multivariable binary regression analysis with the presence of glaucoma as dependent variable and all variables as independent parameter, which were significantly associated with the prevalence of glaucoma in the univariate analysis and which could be regarded to be causally related to glaucoma, instead of being a consequence of it. We then excluded step by step those variables from the list of independent parameters which were no longer significantly associated with the prevalence of glaucoma, starting with the parameters with the highest *P*-values. In a fourth step of the statistical analysis, we added to the list of independent parameters those variables which may have been changed by glaucoma. In a fifth step, we assessed the prevalence of glaucoma as defined by the criteria of the International Society of Geographic and Epidemiological Ophthalmology ISGEO [11]. In that definition, criteria for a category 1 diagnosis (structural and functional evidence) were a vertical cup/disc diameter ratio (VCDR) or an inter-eye asymmetry in the VCDR of ≥97.5th percentile for the normal population, or a neuroretinal rim width reduced to ≤0.1 VCDR (between 11 to 1 o'clock or 5 to 7 o'clock), in addition to a definite visual field defect consistent with glaucoma. Criteria for the category 2 diagnosis (advanced structural damage with unproven visual field loss) were a VCDR or a VCDR asymmetry ≥99.5th percentile for the normal population. Criteria for a category 3 diagnosis (for eyes the optic nerve head of which could not be examined or for which a visual field examination was not possible) were a visual acuity <3/60 combined with either an intraocular pressure >99.5th percentile, or definite glaucoma medical records such as filtering surgery history. Mean ocular perfusion pressure was defined as: 2/3 (diastolic blood pressure + 1/3 x (systolic blood pressure – diastolic blood pressure)) – intraocular pressure.

## Results

Out of the 9422 eyes (4711 subjects), optic disc photographs were available for 8869 (94.1%) eyes of 4570 (97.0%) subjects. The other 141 participants were excluded since assessable photographs of the optic nerve head were not available. The mean age of the 4570 participants (53.8% women) was 48.5±12.9 years (median: 45.0 years; range: 30 – 100 years), with 23.8% being within the age group of 30 to 39 years, 29.2% in the group of 40 to 49 years, 17.1% in the group of 50 to 59 years, 19.1% in the group of 60 to 69 years, and 11.0% in the age group of 70+ years. The mean refractive error was –0.06±1.70 diopters (median: +0.12 diopters; range: –22.0 to +9.38 diopters), and mean axial length was 22.64±0.86 mm (median: 22.61 mm; range: 18.08 to 32.70 mm). The group of subjects without optic disc assessment as compared with the group of subjects included into this study was significantly older (64.6±12.9 years versus 48.5±12.9 years; *P*<0.001), was significantly more myopic (–1.05±2.54 diopters versus –0.06±1.70 diopters; *P*<0.001), had significantly longer axial length (22.87±1.32 mm versus 22.64±0.86 mm; *P*<0.001), and had significantly more women (53.8% versus 49.0%; *P* = 0.03). Intraocular pressure did not vary significantly between both groups (13.8±4.6 mmHg versus 13.8±3.4 mm Hg; *P* = 0.78).

**Figure 1:**
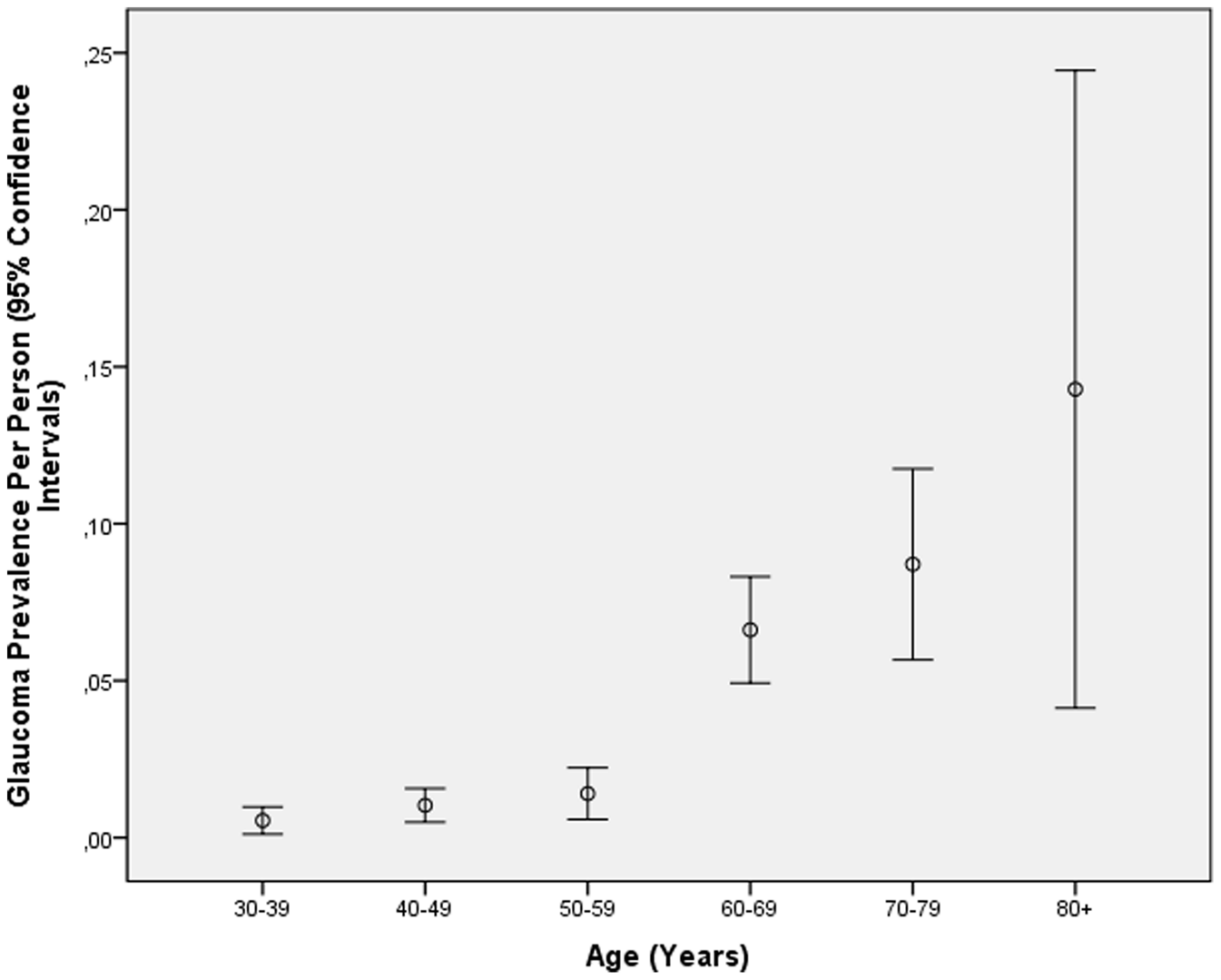
Prevalence of Glaucoma (As defined by Ophthalmoscopy) in the Central India Eye and Medical Study.

Using the optic nerve head criteria for the definition for glaucomatous optic neuropathy, glaucoma was detected in 193 eyes (2.18% (95%CI: 1.87, 2.48) of 122 subjects (51 unilateral, 71 bilateral) (2.67% (95%CI: 2.20, 3.14). In univariate analysis, the glaucoma frequency increased significantly with older age (*P*<0.001). The prevalence of glaucoma per person for the age groups of 30–39 years, 40–49 years, 50–59 years, 60–69 years, 70–79 years, and 80+ years was 0.54% (95%CI: 0.11, 0.98), 1.03% (95%CI: 0.49, 1.57), 1.40% (95%CI: 0.58, 2.23), 6.62% (95%CI: 4.92, 8.31), 8.71% (95%CI: 5.55, 11.75), and 14.3% (95%CI: 4.13, 24.4), respectively (Fig. 1). For the study population aged 40+ years, glaucoma prevalence was 3.45% (95%CI: 2.84, 4.07), for the population aged 50+ years, it was 5.11% (95%CI: 4.14, 6.07), and for the population aged 60+ years, it was 7.50% (95%CI: 6.02, 8.99).

The prevalence of glaucoma was significantly additionally associated with lower body height (*P* = 0.03), lower body weight (*P*<0.001), lower body mass index (*P*<0.001), higher systolic (*P*<0.001) and diastolic (*P*<0.001) blood pressure and prevalence of arterial hypertension (*P*<0.001), lower level of education (*P*<0.001), lower blood concentration of hemoglobin (*P* = 0.03), and with the ocular parameters of higher intraocular pressure (*P*<0.001), higher ocular perfusion pressure (*P* = 0.03), longer axial length (*P*<0.001), more myopic refractive error (*P*<0.001), higher degree of nuclear cataract (*P*<0.001), narrower opening of the anterior chamber angle as assessed by gonioscopy (*P* = 0.001), higher anterior chamber angle pigmentation (*P*<0.001), higher presence of pseudoexfoliation (*P*<0.001), larger optic disc (*P* = 0.001) and optic cup (*P*<0.001), smaller neuroretinal rim (*P* = 0.003), higher horizontal and vertical cup/disc diameter ratios (*P*<0.001), lower retinal nerve fiber layer cross section area as measured by the HRT (*P*<0.001), lower visibility of the macular wall reflex (*P* = 0.006), higher prevalence of myopic retinopathy (*P*<0.001), and higher prevalence of optic disc hemorrhages (*P*<0.001). The prevalence of glaucoma was not significantly associated with the systemic parameters of gender (*P* = 0.07), pulse rate (*P* = 0.10), score of psychic depression (*P* = 0.98), blood concentration of high-density lipoproteins (P = 0.95), cholesterol (*P* = 0.16), creatinine (*P* = 0.98), glycosylated hemoglobin HbA1c (*P* = 0.92), presence of diabetes mellitus (*P* = 0.46), erythrocyte sedimentation rate (*P* = 0.16), drinking alcohol (*P* = 0.19), and with the ocular parameters of anterior corneal curvature (*P* = 0.79), central corneal thickness (*P* = 0.07), anterior chamber depth (*P* = 0.91), lens thickness (*P* = 0.28), prevalence of retinal vein occlusions (*P* = 0.50), presence of central retinal artery occlusion (*P* = 1.00), presence of diabetic retinopathy (*P* = 1.00), presence of early age-related macular degeneration (*P* = 0.34), presence of myelinated retinal nerve fibers (*P* = 0.52).

In a first step of a multivariable analysis, a binary regression analysis was performed with the presence of glaucoma as dependent variable and all the variables as independent parameters, which were significantly associated with the prevalence of glaucoma in the univariate analysis and which could be regarded to be causally related to glaucoma (instead of being a consequence of it, such as a decreased neuroretinal rim area). The list of independent parameters included age, body height, body weight, body mass index, systolic and diastolic blood pressure, level of education, blood concentration of hemoglobin, intraocular pressure, ocular perfusion pressure, axial length, refractive error, degree of nuclear cataract, opening of the anterior chamber angle, anterior chamber angle pigmentation, presence of pseudoexfoliation, optic disc area, prevalence of myopic retinopathy and prevalence of optic disc hemorrhages. We then excluded step by step those variables from the list of independent parameters which were no longer significantly associated with the prevalence of glaucoma, starting with the parameters with the highest *P*-values. After the step-wise exclusion of anterior chamber angle pigmentation (*P* = 0.95), ocular perfusion pressure and degree of nuclear cataract (*P* = 0.66), presence of pseudoexfoliation (*P* = 0.95), systolic blood pressure (*P* = 0.66), diastolic blood pressure (*P* = 0.61), and body height (*P* = 0.13), prevalence of glaucoma remained to be significantly associated with older age (*P*<0.001), lower body mass index (*P* = 0.01), lower blood concentration of hemoglobin (*P* = 0.001), higher intraocular pressure (*P*<0.001), higher mean arterial blood pressure (*P* = 0.002), presence of a disc hemorrhage (*P*<0.001), higher prevalence of myopic retinopathy (*P*<0.001), longer axial length (*P* = 0.004), larger optic disc size (*P*<0.001), lower level of education (*P* = 0.03), and more narrow anterior chamber angle (*P* = 0.01).

We then added to the list of independent parameters those variables which may have been changed by glaucoma. These were the variables of neuroretinal rim area, optic cup area, horizontal and vertical cup/disc diameter ratios, retinal nerve fiber layer cross section area as measured by the HRT, and visibility of the macular wall reflex. We then removed the parameters which showed the least likely association with glaucoma (i.e., the highest *P*-value). After a step-wise removal of the visibility of the macular wall reflex (*P* = 0.99), horizontal cup/disc ratio (*P* = 0.25), optic disc area (*P* = 0.90), optic cup area (*P* = 0.14) and finally neuroretinal area (*P* = 0.08), one arrived at a model in which glaucoma was significantly associated with older age (*P*<0.001), lower body mass index (*P* = 0.025), lower blood concentration of hemoglobin (*P* = 0.03), higher intraocular pressure (*P*<0.001), higher mean blood pressure (*P* = 0.009), presence of a disc hemorrhage (*P*<0.001), higher prevalence of myopic retinopathy (*P*<0.001), lower level of education (*P* = 0.03), longer axial length (*P*<0.001), smaller retinal nerve fiber layer cross section area (*P*<0.001), higher vertical cup/disc diameter ratio (*P*<0.001), and narrowness of the anterior chamber angle (*P* = 0.02) (Table 1). If we added central corneal thickness to this multivariable model, it was not significantly (*P* = 0.69) associated with the glaucoma prevalence.

**Table 1:** Associations between the Prevalence of Glaucoma and Systemic and Ocular Parameters (Multivariable Analysis) in the Central India Eye and Medical Study.

	Regression Coefficient B	*P*-Value	Odds Ratio	95% Confidence Interval of Odds ratio
Age (Years)	0.06	<0.001	1.06	1.04, 1.08
Body Mass Index (kg/m^2^)	–0.08	0.025	0.92	0.86, 0.99
Blood Concentration of Hemoglobin (###)	–0.14	0.027	0.87	0.77, 0.99
Intraocular Pressure (mmHg)	0.16	<0.001	1.17	1.12, 1.23
Mean Blood Pressure (mmHg)	0.02	0.009	1.02	1.01, 1.03
Optic Disc Hemorrhage	5.65	<0.001	283	53, 1514
Presence of Myopic Retinopathy	3.01	<0.001	20.3	3.88, 106
Axial Length (mm)	0.41	<0.001	1.51	1.20, 1.89
Level of Education	–0.25	0.03	0.78	0.62, 0.98
Retinal Nerve Fiber Layer Cross Section Area (####)	–0.96	<0.001	0.38	0.25, 0.58
Vertical Cup/Disc Diameter Ratio	10.2	<0.001	27021	2887, 252902
Anterior Chamber Narrowness (Open, Occludable, Synechial Closure, Appositional Closure)	0.66	0.02	1.94	1.13, 3.33

According to the gonioscopic finding, the whole glaucoma group was differentiated into an open-angle glaucoma group (n = 108 subjects (88.5%); 172 eyes) and subjects with primary angle-closure glaucoma (n = 14 (11.5%); 21 eyes). Prevalence of open-angle glaucoma was 1.93% (95%CI: 1.64, 2.22), and prevalence of angle-closure glaucoma was 0.24% (95%CI: 0.14, 0.34). In multivariable analysis, the prevalence of open-angle glaucoma was significantly associated with the similar parameters as was the whole glaucoma group associated with (Table 2). In contrast, the prevalence of angle-closure glaucoma was significantly correlated with older age (*P* = 0.005), higher intraocular pressure (*P*<0.001), higher prevalence of disc hemorrhages (*P*<0.001), more hyperopic refractive error (*P* = 0.003), shallower anterior chamber (*P* = 0.02), shorter axial length (*P* = 0.01) and higher vertical cup/disc ratio (*P*<0.001), while it was not significantly associated with the level of education (*P* = 0.93), presence of myopic retinopathy (*P* = 0.99), mean blood pressure (*P* = 0.23), blood concentration of hemoglobin (*P* = 0.20), and body mass index (*P* = 0.21) (Table 3).

**Table 2:** Associations between the Prevalence of Open-Angle Glaucoma and Systemic and Ocular Parameters (Multivariable Analysis) in the Central India Eye and Medical Study.

	Regression Coefficient B	*P*-Value	Odds Ratio	95% Confidence Interval of Odds ratio
Age (Years)	0.05	<0.001	1.05	1.03, 1.07
Body Mass Index (kg/m^2^)	–0.10	0.008	0.90	0.84, 0.97
Blood Concentration of Hemoglobin	0.056			
Intraocular Pressure (mmHg)	0.15	<0.001	1.16	1.10, 1.22
Mean Blood Pressure (mmHg)	0.02	0.045	1.02	1.01, 1.03
Optic Disc Hemorrhage	5.04	<0.001	154	34..8, 682
Presence of Myopic Retinopathy	3.11	<0.001	22.5	4.28, 118
Axial Length (mm)	0.37	0.002	1.45	1.15, 1.83
Level of Education	–0.34	0.006	0.71	0.56, 0.91
Retinal Nerve Fiber Layer Cross Section Area	–1.01	<0.001	0.37	0.24, 0.56
Vertical Cup/Disc Diameter Ratio	10.0	<0.001	22939	2293, 229407

**Table 3:** Associations between the Prevalence of Angle-Closure Glaucoma and Systemic and Ocular Parameters (Multivariable Analysis) in the Central India Eye and Medical Study.

	Regression Coefficient B	*P*-Value	Odds Ratio	95% Confidence Interval of Odds ratio
Age (Years)	0.07	0.005	1.07	1.02, 1.13
Intraocular Pressure (mmHg)	0.15	<0.001	1.16	1.07, 1.26
Optic Disc Hemorrhage	3.87	<0.001	48.0	4.89, 471
Refractive Error (Diopters)	–0.33	0.003	0.72	0.58, 0.90
Anterior Chamber Depth (mm)	–1.71	0.02	0.18	0.05, 0.73
Axial Length (mm)	–0.70	0.01	0.50	0.28, 0.87
Vertical Cup/Disc Ratio	9.07	<0.001	8696	67, 1135517

In the whole study population, cataract surgery had been performed in 273 eyes. Within this group of eyes after cataract surgery, 37 (13.6%) eyes were classified to have glaucoma, with 34 (92%) subject having open-angle glaucoma and 3 (8%) eyes having angle-closure glaucoma. This ratio of 11 to 1 was higher than the ratio in the whole study population (108/14 or 7 to 1).

According to the World Health Organization criteria, low vision and blindness were defined as visual acuity in the better seeing eye as <20/60 to 20/400, and as <20/400, respectively. In the 108 subjects with open-angle glaucoma, 34 (31.5%) subjects had low vision due to glaucoma, and one (0.9%) of the subjects with open-angle glaucoma was blind. Unilateral visual impairment was present in 65 (37.8%) of the 172 eyes with open-angle glaucoma, and glaucoma related blindness occurred in 18 (10.5%) eyes with open-angle glaucoma. Out of 14 subjects with primary angle-closure glaucoma, 3 (21%) subjects had low vision and none of the subjects was blind due to glaucoma. Unilateral visual impairment was present in 7 (33%) of the 21 eyes with angle-closure glaucoma, and glaucoma related blindness occurred in 4 (19%) eyes with angle-closure glaucoma.

The mean vertical cup/disc diameter ratio was 0.55±0.12 (median: 0.56) (Table 4). The 97.5th percentile of the vertical cup/disc ratio was 0.74, and the 99.5th percentile was 0.81 (Table 1). The mean inter-eye asymmetry of the vertical cup/disc ratio was 0.08±0.08, with the 97.5th percentile at 0.30 and the 99.5th percentile at 0.52. The mean intraocular pressure was 13.8±3.5 mmHg, with the 97.5th percentile at 20 mmHg. A visual field defect defined as sensitivity loss at any location was found for 2097 eyes (25.7%) subjects.

**Table 4:** Definition of Glaucoma by the International Society of Geographical and Epidemiological Ophthalmology (ISGEO) and the Number of Subjects of the Beijing Eye Study in Each Category.

Category	Definition	Number of Eyes
Category 1	definition 1AVCDR ≥ 97.5th percentile + VFD	50
definition 1B	VCDR Asymmetry ≥ 97.5th Percentile + VFD	21
definition 1C	Rim Width ≤ 0.1VCDR + VFD	41
Category 2	definition 2AVCDR ≥ 99.5th percentile	42
definition 2B	VCDR Asymmetry ≥ 99.5th Percentile	20
Category 3	definition 3Visual acuity <3/60 + IOP>99.5th Percentile	16
	/ filtering surgery	
Glaucoma	Total	138

VCDR: Vertical cup/disc ratio.

VFD: Visual field defect.

IOP: Intraocular pressure.

The total sum of subjects adds up to more than the real total number of glaucomatous eye, since there was an overlap between the categories or definitions within each category.

**Figure 2:**
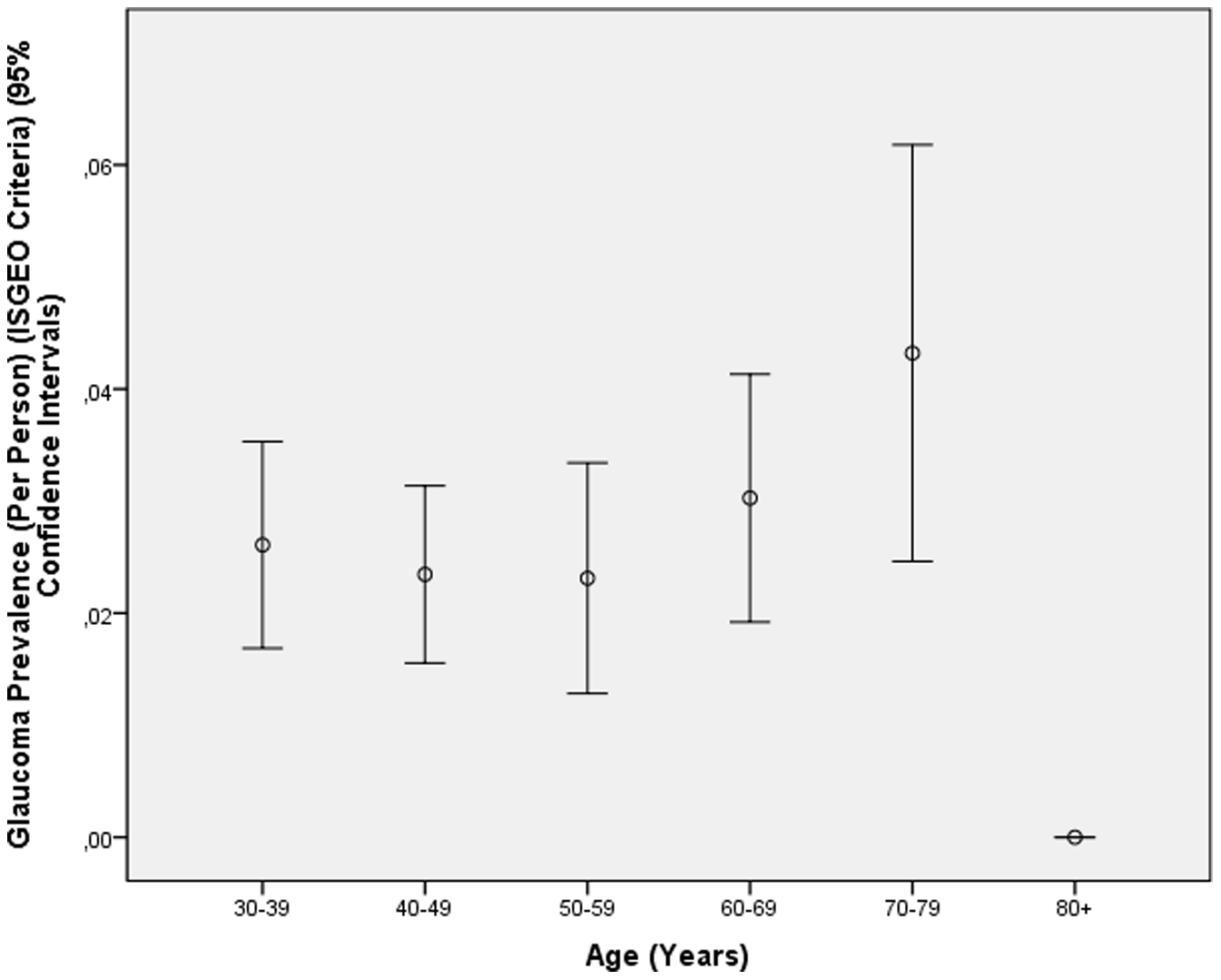
Prevalence of Glaucoma (As Defined by the Criteria of the International Society of Geographical and Epidemiological Ophthalmology ISGEO) in the Central India Eye and Medical Study.

Using the criteria of the International Society of Geographical and Epidemiological Ophthalmology, glaucoma was detected in 138 eyes (1.61±0.14% (95%CI: 1.34, 1.87) of 130 subjects (122 unilateral, 8 bilateral) (prevalence: 2.8%±0.2% (95% CI: 2.3, 3.3). The number of subjects in each diagnostic category is listed in Table 4. Out of the 138 eyes with ISGEO glaucoma, 123 (89.1%) fulfilled the criteria of OAG. Out of the 138 eyes with ISGEO glaucoma, 26 (18.8%) were considered to be glaucomatous by ophthalmoscopy. Out of the 193 eyes with the ophthalmoscopic glaucoma diagnosis, 26 (13.5%) eyes fulfilled the ISGEO criteria for glaucoma. In contrast to the prevalence of glaucoma as defined by ophthalmoscopy, the prevalence of glaucoma as defined by the ISGEO criteria was not significantly (*P* = 0.25) associated with higher age (Fig. 2). In view of the missing association between ISGEO glaucoma prevalence and age in contrast to the clear association of ophthalmoscopic glaucoma prevalence and age, the prevalence of ISGEO glaucoma was not further elaborated in this study.

## Discussion

In our population-based in rural Central India, glaucoma was detected in 2,67% of the subjects, in whom open angle glaucoma (OAG) and primary angle closure glaucoma (PACG) accounted for 1.93% and 0.24%. In multivariable analysis, glaucoma was associated with older age, lower body mass index, lower blood concentration of hemoglobin, higher intraocular pressure, higher mean blood pressure, presence of a disc hemorrhage, higher prevalence of myopic retinopathy, lower level of education, longer axial length, smaller retinal nerve fiber layer cross section area, higher vertical cup/disc diameter ratio, and narrowness of the anterior chamber angle (Table 1).

For India, the frequency of glaucoma has been reported by the Vellore Eye Survey, Andhra Pradesh Eye Disease Study, Aravind Comprehensive Eye Survey, Chennai Glaucoma Study, and West Bengal Glaucoma Study [20]–[27]. Most of these studies were carried out in urban or semi-urban environments, while a markedly rural population was rarely examined. The prevalence of 3.45% of glaucoma in our study population aged 40+ years was similar to the glaucoma prevalence in the South Indian Chennai Glaucoma Study in which in the study population aged 40+ years the prevalence of open-angle glaucoma was 3.51% (95%CI: 3.04, 4.0) and the prevalence of angle-closure glaucoma was 0.88% (95%CI: 0.60, 1.16) [20], [21]. In the South Indian Aravind Eye Survey, the prevalence of glaucoma in the population aged 40+ years was 2.6% (95%CI: 2.2, 3.0) with a ratio of open-angle glaucoma to angle-closure glaucoma of 1.7% to 0.5% or 3.4 : 1 [22]. In the South Indian Andhra Pradesh Eye Disease Study, the ratio of the prevalence open-angle glaucoma and suspected open-angle glaucoma to the prevalence of angle-closure glaucoma was 2.41% to 0.71% or 3.4 to 1 [23]–[26]. In the Indian West Bengal Glaucoma Study, prevalence of glaucoma in the study population aged 50+ years was 2.7% (95% CI: 1.7 to 3.7) with a ratio of 10 : 1 of open-angle glaucoma to angle-closure glaucoma [23]. The glaucoma prevalence in our study population was slightly higher than in the recent Singapore Indian eye study which examined 3400 persons aged 40 to 80 years [28]. Using the ISGEO criteria. The glaucoma prevalence was 2.29% in the study population, leading to an age-standardized prevalence of 1.95% (95%CI: 1.5%–2.5%). The ratio of primary open-angle glaucoma to primary angle-closure glaucoma was 1.25% to 0.12% or 10.4 to 1. The glaucoma prevalence in our study population was also similar to the glaucoma prevalence of 3.8% reported in the Liwan Eye Study in Guangzhou/South China [29], and it is similar to the glaucoma prevalence of 3.7% (95%CI: 3.1, 4.2) in the North Chinese Beijing Eye Study [30]. In other investigations [31]–[43], the reported glaucoma prevalence ranged between 0.5% in a rural population in Mongolia [31], 1.6% in a Singaporean urban population [32], 1.7% in Melbourne/Australia [33], 2.1% in a rural population in the United States of America [34], 2.7% in South Africa [35], 3.1% in rural East Africa [36], 5.7% in a study population of 760 people aged 65–74 years in Central Sweden [37], and 7.0% in a black population group in Barbados [38]. One of the reasons for the discrepancies between the studies may be ethnic differences with a presumably relatively high rate of glaucoma in the African population groups.

Our study showed a ratio of open-angle glaucoma to angle-closure glaucoma of 108/14 or 7.7:1. This ratio is similar to the ratio reported in some other Asian studies such as the Japanese Tajimi study (3.9% to 0.6% or 6.5:1) [43], and in the Singapore Malay Eye Study (2.5% to 0.1% (25:1) [42]. It is higher than in the South Indian Chennai Glaucoma Study (3.51% : 0.81% or 4:1) [20], [21], in the South Indian Aravind Eye Survey (3.4:1) [22], and in the Tanjong Pagar study (2.4% to 0.8% or 3 : 1) [32]. The ratio in our study was markedly higher than in the Liwan Study from South China (1.4 to 1) or in the Beijing Eye Study (2.6 to 1) [29], [30].

In multivariable analysis, glaucoma prevalence increased significantly with older age (*P*<0.001), lower body mass index (*P* = 0.025), lower blood concentration of hemoglobin (*P* = 0.03), higher intraocular pressure (*P*<0.001), higher mean blood pressure (*P* = 0.009), presence of a disc hemorrhage (*P*<0.001), higher prevalence of myopic retinopathy (*P*<0.001), lower level of education (*P* = 0.03), longer axial length (*P*<0.001), smaller retinal nerve fiber layer cross section area (*P*<0.001), higher vertical cup/disc diameter ratio (*P*<0.001), and narrowness of the anterior chamber angle (*P* = 0.02) (Table 1). The age-related increase of the glaucoma prevalence agrees with all preceding investigations showing a similar increase with age [1], [4]–[6], [20]–[43]. In a similar manner, the associations between intraocular pressure and glaucoma and between myopia and glaucoma have been reported in previous population-based studies [20]–[43]. The association of a higher prevalence of glaucoma with lower body mass index confirms previous investigations in which a lower body mass index was associated with a higher prevalence and incidence of glaucoma [44]–[46]. Pasquale and colleagues assessed the relationship between anthropometric measures and incident primary open-angle glaucoma in 78,777 women of the Nurses' Health Study and in 41,352 men of the Health Professionals Follow-up Study [44]. They found that among women, a higher body mass index was associated with a lower risk of primary open-angle glaucoma with intraocular pressure readings of 21 mmHg or less at diagnosis. In the Barbados Eye Study, persons most likely to have open-angle glaucoma were older men and had a family history of open-angle glaucoma, high intraocular pressure, lean body mass, and cataract history [1]. In a similar manner, Zheng and colleagues in the Singapore Malay Eye Study and Xu and colleagues in the Beijing Eye Study found that persons who were taller or had lower body mass index had a smaller neuroretinal rim area and a larger optic cup-to-disc area ratio [45], [46]. Since body mass index is correlated with cerebrospinal fluid pressure, our finding supports the notion that a low cerebrospinal fluid pressure may in some patients play a role in the pathogenesis of glaucomatous optic neuropathy [47], [48].

Central corneal thickness was associated with the glaucoma prevalence neither in univariate analysis (*P* = 0.07) nor in multivariable analysis (*P* = 0.69). Since most of the glaucoma patients in our study population were not aware of their disease, a confounding factor may be unlikely. It suggests that central corneal thickness may not be related to glaucoma susceptibility, what agrees with, or is contradictory to some previous studies [3], [28], [49], [50].

Presence of myopic retinopathy and in addition, long axial length were factors associated with the glaucoma prevalence. These findings confirm results from previous studies on other populations in which high myopia was a highly significant risk factor for glaucomatous optic neuropathy [51]. The association between glaucoma prevalence and the presence of myopic retinopathy was in addition to the association between glaucoma prevalence and longer axial length (or myopic refractive error) as also reported in previous studies such as the Early Manifest Glaucoma Trial [52]. In a similar manner, our population-based study in a remote rural region confirms the strong association of optic disc hemorrhages with glaucomatous optic neuropathy.

In our study, the glaucoma definition based on the subjective assessment as compared to the definition based on the ISGEO criteria of the optic nerve head appearance resulted in apparently more reasonable results. In many recent population-based investigations on the prevalence of glaucoma such as the Liwan Eye Study the ISGEO definition of glaucomatous optic neuropathy has usually been applied, based on a consensus published by Foster and colleagues [11], [29]. The clear advantages of such a consensus definition are that it is the first commonly used definition of glaucomatous optic neuropathy and that it thus makes the results of different population-based studies on the prevalence of glaucoma comparable. Using the quantitative parameters for glaucoma diagnosis as recommended by the ISGEO criteria harbors, however, certain limitations as also discussed by Foster and colleagues. First, the dependence of the cup/disc diameter ratios and of the neuroretinal rim width on the optic disc size was not taken into account [53], [54]. It can lead to an over-diagnosis of glaucoma in eyes with abnormally large, otherwise normal, optic discs (“pseudo-glaucomatous but physiological macro-cups in primary macro-discs”), and to an underdiagnosis of glaucoma in eyes with small optic discs (“pseudo-normal, however, glaucomatous mini-cups in mini-discs”) [55]. Correspondingly, hospital-based studies have shown that correcting the vertical cup/disc diameter ratio for its dependence on the disc size significantly improves the diagnostic precision to detect glaucoma [56]. Second, hospital-based investigations have shown that the diagnostic precision to detect glaucoma was lower for quantitative optic nerve head parameters such as the cup/disc diameter ratios or neuroretinal rim width measurements than for qualitative parameters such as the so-called Inferior-Superior- Nasal- Temporal-rule (ISNT rule) of the normal configuration of the neuroretinal rim, and the presence or absence of localized retinal nerve fiber layer defects [57], [58]. Third, the limitations of the ISGEO criteria may also be suggested by the use of a cut-off criterion of a 97.5th percentile for a quantitative parameter such as the VCDR, since without applying additional criteria (like the presence of definite visual field defect consistent with glaucoma as applied in the ISGEO criteria), the prevalence of the disease would be approximately 2.5%. Interestingly, most population-based studies using the ISGEO definition of glaucoma arrived at a glaucoma prevalence of about 2–3%. Without doubt, the consensus based definition of a disease for which none comparable definition was previously been available, is a major step forward which allows comparing the prevalence data between studies on various populations. In addition, using a quantitative parameter allows a “machine-driven” diagnosis without inter-observer variability. One may, however, also take into account the limitations of such a definition and wonder whether the additional inclusion of qualitative variables such as those described above and whether considering the dependence of quantitative disc parameters on the disc size would not further improve the validity of glaucoma prevalence figures found in population-based studies.

Potential limitations of our study should be discussed. First, a major concern in any prevalence study is nonparticipation. The Central India Eye and Medical Study had a reasonable response rate of 80.1%, however, differences between participants and non-participants can lead to a selection artifact. Second, our study included only those who resided in a purely rural region, a region that can be considered to be markedly rural based on responses to the questionnaire regarding socioeconomic background and lifestyle. The study did not include subjects from an urban region, so we can provide no information on any differences between rural and urban regions with respect to the examined parameters. Third, our study as cross-section investigation does not allow firm statements on a longitudinal association, i.e. temporal causation cannot be established with the current cross-sectional design. Fourth, it could not be determined whether disk hemorrhages were associated with glaucoma or were a result of posterior vitreous detachment or other vascular pathology [59]. Fourth, many factors were primarily included into the statistical analysis ranging from education level to body mass index, so that just by chance one of them may have exhibited a statically significant association with glaucoma. In an advanced step of the statistical analysis however, we performed a multivariable statistical analysis in which the inter-dependencies between the independent parameters were taken into account. Fifth, frequency doubling threshold perimetry was used to screen for visual field defects, although its results are sensitive to lens opacifications. One may also wonder how well our rural and technology-naïve study population performed their perimetric examinations so that the prevalence of visual field defects may have been overestimated. Sixth, the ISNT rule as a major part in the definition of glaucomatous optic neuropathy based on the ophthalmoscopical assessment of the optic nerve head. Yet there are several studies indicating that the ISNT rules are non-specific in some clinical situations [60]–[63]. Seventh, due to the relatively large number of study participants, a problem of mass significance may be present. This problem may however been solved by the relatively low P-values for the associations of glaucoma prevalence with most discussed parameters so that a Bonferroni correction would not have led to a different result. In addition, the odds ratios described the impact of the individual parameter on the prevalence of glaucoma. Eighth, the definition of a visual field defect was a defect in the frequency doubling perimetry. This definition is not at all equal to the definition of a glaucomatous visual field defect as used in hospital-based studies. It is however, not easily feasible to perform a standard computerized static perimetry under the conditions of a population-based investigation. Strengths of our study included that first, the population size was relatively large; second, that the study population lived in remote rural villages in Central India where modern civilization had not had yet a marked influence on daily life; and third, that in contrast to some previous population-based studies, persons with an age between 30 and 40 years were included.

In conclusion, glaucoma prevalence in remote rural Central India was comparable to other regions. It increased with older age, lower body mass index, lower blood concentration of hemoglobin, higher intraocular pressure, presence of an optic disc hemorrhage, higher prevalence of myopic retinopathy, lower level of education, longer axial length, smaller retinal nerve fiber layer cross section area, and higher vertical cup/disc diameter ratio. The ratio of open-angle glaucoma to angle-closure glaucoma was about 8:1.

## References

[1] Leske MC, Connell AM, Wu SY, Hyman LG, Schachat AP (1995) Risk factors for open-angle glaucoma. The Barbados Eye Study. Arch Ophthalmol 113: 918–924. 7605285 10.1001/archopht.1995.01100070092031

[2] Mitchell P, Hourihan F, Sandbach J, Wang JJ (1999) The relationship between glaucoma and myopia: the Blue Mountains Eye Study. Ophthalmology 106: 2010–2015. 10519600 10.1016/s0161-6420(99)90416-5

[3] Brandt JD, Beiser JA, Kass MA, Gordon MO (2001) Central corneal thickness in the Ocular Hypertension Treatment Study (OHTS). Ophthalmology 108: 1779–1788. 11581049 10.1016/s0161-6420(01)00760-6

[4] Nouri-Mahdavi K, Hoffman D, Coleman AL, Liu G, Li G (2004) Advanced Glaucoma Intervention Study. Predictive factors for glaucomatous visual field progression in the Advanced Glaucoma Intervention Study. Ophthalmology 111: 1627–1635. 15350314 10.1016/j.ophtha.2004.02.017

[5] Musch DC, Gillespie BW, Lichter PR, Niziol LM, Janz NK, et al (2009) Visual field progression in the Collaborative Initial Glaucoma Treatment Study the impact of treatment and other baseline factors. Ophthalmology 116: 200–207. 19019444 10.1016/j.ophtha.2008.08.051 PMC3316491

[6] Heijl A, Leske MC, Bengtsson B, Hyman L, Bengtsson B, et al (2001) Reduction of intraocular pressure and glaucoma progression: results from the Early Manifest Glaucoma Trial. Arch Ophthalmol 120: 1268–1279. 10.1001/archopht.120.10.1268 12365904

[7] Nangia V, Jonas JB, Sinha A, Matin A, Kulkarni M, et al (2010) Ocular axial length and its associations in an adult population of Central Rural India. The Central India Eye and Medical Study. Ophthalmology 117: 1360–1366. 20363029 10.1016/j.ophtha.2009.11.040

[8] Jonas JB, Nangia V, Matin A, Joshi P, Ughade S (2010) Prevalence, awareness, control and associations of arterial hypertension in a rural Central India population. The Central India Eye and Medical Study. Am J Hypertension 23: 347–350. 10.1038/ajh.2009.276 20094037

[9] Van Herick W, Shaffer RN, Schwartz A (1969) Estimation of width of angle of anterior chamber. Incidence and significance of the narrow angle. Am J Ophthalmol 68: 626–629. 5344324 10.1016/0002-9394(69)91241-0

[10] Foster PJ, Devereux JG, Alsbirk PH, Lee PS, Uranchimeg D, et al. (2000) Detection of gonioscopically occludable angles and primary angle closure glaucoma by estimation of limbal chamber depth in Asians: modified grading scheme. Br J Ophthalmol 84: ;186–192. 10.1136/bjo.84.2.186 PMC1723375 10655196

[11] Foster PJ, Buhrmann R, Quigley H, Johnson GJ (2002) The definition and classification of glaucoma in prevalence surveys. Br J Ophthalmol 86: 238–242. 11815354 10.1136/bjo.86.2.238 PMC1771026

[12] Thomas R, Sekhar GC, Parikh R (2007) Primary angle closure glaucoma: a developing world perspective. Clin Experiment Ophthalmol 35: 374–378. 17539793 10.1111/j.1442-9071.2007.01489.x

[13] Krishnadas R, Nirmalan PK, Ramakrishnan R, Thulasiraj RD, Katz J, et al (2003) Pseudoexfoliation in a rural population of southern India: the Aravind Comprehensive Eye Survey. Am J Ophthalmol 2003 135: 830–837. 10.1016/s0002-9394(02)02271-7 12788123

[14] Age-Related Eye Disease Study Research Group (2001) The Age-Related Eye Disease Study (AREDS) system for classifying cataracts from photographs: AREDS report no. 4. Am J Ophthalmol 131: 167–175. 11228291 10.1016/s0002-9394(00)00732-7 PMC2032014

[15] Thomas R (2012) Glaucoma in developing countries. Indian J Ophthalmol 60: 446–450. 22944757 10.4103/0301-4738.100546 PMC3491273

[16] Thomas R (2011) Glaucoma in India: current status and the road ahead. Indian J Ophthalmol 59 Suppl:S3-4 10.4103/0301-4738.73678 PMC3038493 21150031

[17] Vongphanit J, Mitchell P, Wang JJ (2002) Prevalence and progression of myopic retinopathy in an older population. Ophthalmology 109: :704 –711. 10.1016/s0161-6420(01)01024-7 11927427

[18] Curtin BJ (1977) The posterior staphyloma of pathologic myopia. Trans Am Ophthalmol Soc 75: 67–86. 613534 PMC1311542

[19] Steidl SM, Pruett RC (1977) Macular complications associated with posterior staphyloma. Am J Ophthalmol 123: 181–187. 10.1016/s0002-9394(14)71034-7 9186123

[20] Vijaya L, George R, Arvind H, Baskaran M, Ve Ramesh S, et al (2008) Prevalence of primary angle-closure disease in an urban south Indian population and comparison with a rural population. The Chennai Glaucoma Study. Ophthalmology 115: 655–660. 17869343 10.1016/j.ophtha.2007.05.034

[21] Vijaya L, George R, Baskaran M, Arvind H, Raju P, et al (2008) Prevalence of primary open-angle glaucoma in an urban south Indian population and comparison with a rural population. The Chennai Glaucoma Study. Ophthalmology 115: 648–654. 17664010 10.1016/j.ophtha.2007.04.062

[22] Ramakrishnan R, Nirmalan PK, Krishnadas R, Thulasiraj RD, Tielsch JM, et al (2003) Glaucoma in a rural population of southern India: the Aravind comprehensive eye survey. Ophthalmology 110: 1484–1490. 12917161 10.1016/S0161-6420(03)00564-5

[23] Garudadri C, Senthil S, Khanna RC, Sannapaneni K, Rao HB (2010) Prevalence and risk factors for primary glaucomas in adult urban and rural populations in the Andhra Pradesh Eye Disease Study. Ophthalmology 117: 1352–1359. 20188420 10.1016/j.ophtha.2009.11.006

[24] Dandona L, Dandona R, Mandal P, Srinivas M, John RK, et al (2000) Angle-closure glaucoma in an urban population in southern India. The Andhra Pradesh eye disease study. Ophthalmology 107: 1710–1716. 10964834 10.1016/s0161-6420(00)00274-8

[25] Dandona L, Dandona R, Srinivas M, Mandal P, John RK, et al (2000) Open-angle glaucoma in an urban population in Southern India: the Andhra Pradesh eye disease study. Ophthalmology 107: 1702–1709. 10964833 10.1016/s0161-6420(00)00275-x

[26] Raychaudhuri A, Lahiri SK, Bandyopadhyay M, Foster PJ, Reeves BC, et al (2005) A population based survey of the prevalence and types of glaucoma in rural West Bengal: the West Bengal Glaucoma Study. Br J Ophthalmol 89: 1559–1564. 16299129 10.1136/bjo.2005.074948 PMC1772964

[27] Jacob A, Thomas R, Koshi SP, Braganza A, Muliyil J (1998) Prevalence of primary glaucoma in an urban south Indian population. Indian J Ophthalmol 46: 81–86. 9847479

[28] Narayanaswamy A, Baskaran M, Zheng Y, Lavanya R, Wu R, et al (2013) The prevalence and types of glaucoma in an urban Indian population: the Singapore Indian eye study. Invest Ophthalmol Vis Sci 10 54: 4621–4627. 10.1167/iovs.13-11950 23745009

[29] He M, Foster PJ, Ge J, Huang W, Zheng Y, et al (2006) Prevalence and clinical characteristics of glaucoma in adult Chinese: a population-based study in Liwan District, Guangzhou. Invest Ophthalmol Vis Sci 47: 2782–2788. 16799014 10.1167/iovs.06-0051

[30] Wang YX, Xu L, Yang H, Jonas JB (2010) Prevalence of glaucoma in North China. The Beijing Eye Study. Am J Ophthalmol 150: 917–924. 20970107 10.1016/j.ajo.2010.06.037

[31] Foster PJ, Baasanhu J, Alsbirk PH, Munkhbayar D, Uranchimeg D, et al (1996) Glaucoma in Mongolia. A population-based survey in Hövsgöl province, northern Mongolia. Arch Ophthalmol 114: 1235–1241. 8859083 10.1001/archopht.1996.01100140435011

[32] Foster PJ, Oen FT, Machin D, Ng TP, Devereux JG, et al (2000) The prevalence of glaucoma in Chinese residents of Singapore: a cross-sectional population survey of the Tanjong Pagar district. Arch Ophthalmol 118: 1105–1111. 10922206 10.1001/archopht.118.8.1105

[33] Wensor MD, McCarty CA, Stanislavsky YL, Livingston PM, Taylor HR (1998) The prevalence of glaucoma in the Melbourne Visual Impairment Project. Ophthalmology 105: 733–739. 9544649 10.1016/S0161-6420(98)94031-3

[34] Klein BE, Klein R, Sponsel WE, Franke T, Cantor LB, et al (1992) Prevalence of glaucoma. The Beaver Dam Eye Study. Ophthalmology 99: 1499–1504. 1454314 10.1016/s0161-6420(92)31774-9

[35] Rotchford AP, Johnson GJ (2002) Glaucoma in Zulus: a population-based cross-sectional survey in a rural district in South Africa. Arch Ophthalmol 120: 471–478. 11934321 10.1001/archopht.120.4.471

[36] Buhrmann RR, Quigley HA, Barron Y, West SK, Oliva MS, et al (2000) Prevalence of Glaucoma in a rural East African population. Invest Ophthalmol Vsi Sci 41: 40–48. 10634599

[37] Ekström C (1996) Prevalence of open-angle glaucoma in central Sweden. Acta Ophthalmol Scand 74: 107–112. 8739672 10.1111/j.1600-0420.1996.tb00052.x

[38] Leske MC, Connell AM, Schachat AP, Hyman L (1994) The Barbados Eye Study. Prevalence of open angle glaucoma. Arch Ophthalmol 112: 821–829. 8002842 10.1001/archopht.1994.01090180121046

[39] Dielemans I, Vingerling JR, Wolfs RC, Hofman A, Grobbee DE, et al (1994) The prevalence of primary open-angle glaucoma in a population-based study in The Netherlands. The Rotterdam Study. Ophthalmology 101: 1851–1855. 7800368 10.1016/s0161-6420(94)31090-6

[40] Varma R, Ying-Lai M, Francis BA, Nguyen BB, Deneen J, et al (2004) Prevalence of open-angle glaucoma and ocular hypertension in Latinos: the Los Angeles Latino Eye Study. Ophthalmology 111: 1439–1448. 15288969 10.1016/j.ophtha.2004.01.025

[41] Bourne RR, Sukudom P, Foster PJ, Tantisevi V, Jitapunkul S, et al (2003) Prevalence of glaucoma in Thailand: a population-based survey in Rom Klao district, Bangkok. Br J Ophthalmol 87: 1069–1074. 12928267 10.1136/bjo.87.9.1069 PMC1771843

[42] Shen SY, Wong TY, Foster PJ, Loo JL, Rosman M, et al (2008) The prevalence and types of glaucoma in Malay people: the Singapore Malay eye study. Invest Ophthalmol Vis Sci 49: 3846–3851. 18441307 10.1167/iovs.08-1759

[43] Yamamoto T, Iwase A, Araie M, Suzuki Y, Abe H, et al (2005) The Tajimi Study report 2: prevalence of primary angle closure and secondary glaucoma in a Japanese population. Ophthalmology 112: 1661–1669. 16111758 10.1016/j.ophtha.2005.05.012

[44] Pasquale LR, Willett WC, Rosner BA, Kang JH (2010) Anthropometric measures and their relation to incident primary open-angle glaucoma. Ophthalmology 117: 1521–1519. 20382429 10.1016/j.ophtha.2009.12.017 PMC2904416

[45] Zheng Y, Cheung CY, Wong TY, Mitchell P, Aung T (2010) Influence of height, weight, and body mass index on optic disc parameters. Invest Ophthalmol Vis Sci 51: 2998–3002. 20071668 10.1167/iovs.09-4470

[46] Xu L, Wang YX, Wang S, Jonas JB (2012) Neuroretinal rim area and body mass index. PLoS One 7: e30104. 22253892 10.1371/journal.pone.0030104 PMC3253809

[47] Ren R, Wang N, Zhang X, Tian G, Jonas JB, et al (2012) Cerebrospinal fluid pressure correlated with body mass index. Graefes Arch Clin Exp Ophthalmol 250: 445–446. 21814821 10.1007/s00417-011-1746-1

[48] Ren R, Jonas JB, Tian G, Zhen Y, Ma K, et al (2010) Cerebrospinal fluid pressure in glaucoma. A prospective study. Ophthalmology 117: 259–266. 19969367 10.1016/j.ophtha.2009.06.058

[49] Chauhan BC, Mikelberg FS, Balaszi AG, LeBlanc RP, Lesk MR, et al (2008) Canadian Glaucoma Study: 2. risk factors for the progression of open-angle glaucoma. Arch Ophthalmol 126: 1030–1036. 18695095 10.1001/archopht.126.8.1030

[50] Leske MC, Heijl A, Hyman L, Bengtsson B, Dong L, et al (2007) Predictors of long-term progression in the early manifest glaucoma trial. Ophthalmology 114: 1965–1972. 17628686 10.1016/j.ophtha.2007.03.016

[51] Xu L, Wang Y, Wang S, Jonas JB (2007) High myopia and glaucoma susceptibility. The Beijing Eye Study. Ophthalmology 114: 216–220. 17123613 10.1016/j.ophtha.2006.06.050

[52] Grødum K, Heijl A, Bengtsson B (2001) Refractive error and glaucoma. Acta Ophthalmol Scand 79: 560–566. 11782219 10.1034/j.1600-0420.2001.790603.x

[53] Bengtsson B (1980) The alteration and asymmetry of cup and disc diameters. Acta Ophthalmol 58: 726–732. 7211261 10.1111/j.1755-3768.1980.tb06685.x

[54] Jonas JB, Gusek GC, Naumann GO (1988) Optic disc, cup and neuroretinal rim size, configuration, and correlations in normal eyes. Invest Ophthalmol Vis Sci 29: 1151–1158. 3417404

[55] Jonas JB, Zäch FM, Gusek GC, Naumann GO (1989) Pseudoglaucomatous physiologic large cups. Am J Ophthalmol 107: 137–144. 2913807 10.1016/0002-9394(89)90212-2

[56] Jonas JB, Bergua A, Schmitz-Valckenberg P, Papastathopoulos KI, Budde WM (2000) Ranking of optic disc variables for detection of glaucoma damage. Invest Ophthalmol Vis Sci 41: 1764–1773. 10845597

[57] Jonas JB, Budde WM, Lang P (1998) Neuroretinal rim width ratios in morphological glaucoma diagnosis. Br J Ophthalmol 82: 1366–1371. 9930265 10.1136/bjo.82.12.1366 PMC1722465

[58] Jonas JB, Budde WM, Panda-Jonas S (1999) Ophthalmoscopic evaluation of the optic nerve head. Surv Ophthalmol 43: 293–320. 10025513 10.1016/s0039-6257(98)00049-6

[59] Jonas JB, Ritch R (2012) Optic disc hemorrhage and posterior vitreous hemorrhage. Clin Exp Ophthalmol 40: e116–117. 22044654 10.1111/j.1442-9071.2011.02731.x

[60] Morgan JE, Bourtsoukli I, Rajkumar KN, Ansari E, Cunliffe IA, et al (2012) The accuracy of the inferior>superior>nasal>temporal neuroretinal rim area rule for diagnosing glaucomatous optic disc damage. Ophthalmology 119: 723–730. 22365059 10.1016/j.ophtha.2011.10.004

[61] Sihota R, Srinivasan G, Dada T, Gupta V, Ghate D, et al (2008) Is the ISNT rule violated in early primary open-angle glaucoma—a scanning laser tomography study. Eye 22: 819–824. 17435693 10.1038/sj.eye.6702798

[62] Pogrebniak AE, Wehrung B, Pogrebniak KL, Shetty RK, Crawford P (2010) Violation of the ISNT rule in Nonglaucomatous pediatric optic disc cupping. Invest Ophthalmol Vis Sci 51: 890–895. 19737890 10.1167/iovs.09-3837

[63] Harizman N, Oliveira C, Chiang A, Tello C, Marmor M, et al (2006) The ISNT rule and differentiation of normal from glaucomatous eyes. Arch Ophthalmol 124: 1579–1583. 17102005 10.1001/archopht.124.11.1579

